# Using Continuous Glucose Monitoring to Prescribe a Time to Exercise for Individuals with Type 2 Diabetes

**DOI:** 10.3390/jcm12093237

**Published:** 2023-04-30

**Authors:** Courtney R. Chang, Brooke M. Russell, Tannia Cyriac, Monique E. Francois

**Affiliations:** 1 School of Medical, Indigenous and Health Sciences, Faculty of Science, Medicine and Health, University of Wollongong, Wollongong, NSW 2522, Australia; 2 Illawarra Health and Medical Research Institute, University of Wollongong, Wollongong, NSW 2522, Australia

**Keywords:** prescribed exercise timing, type 2 diabetes, continuous glucose monitoring, hyperglycaemia, physical activity

## Abstract

This study examines the potential utility of using continuous glucose monitoring (CGM) to prescribe an exercise time to target peak hyperglycaemia in people with type 2 diabetes (T2D). The main aim is to test the feasibility of prescribing an individualised daily exercise time, based on the time of CGM-derived peak glucose, for people with T2D. Thirty-five individuals with T2D (HbA1c: 7.2 ± 0.8%; age: 64 ± 7 y; BMI: 29.2 ± 5.2 kg/m^2^) were recruited and randomised to one of two 14 d exercise interventions: i) ExPeak (daily exercise starting 30 min before peak hyperglycaemia) or placebo active control NonPeak (daily exercise starting 90 min after peak hyperglycaemia). The time of peak hyperglycaemia was determined via a two-week baseline CGM. A CGM, accelerometer, and heart rate monitor were worn during the free-living interventions to objectively measure glycaemic control outcomes, moderate-to-vigorous intensity physical activity (MVPA), and exercise adherence for future translation in a clinical trial. Participation in MVPA increased 26% when an exercise time was prescribed compared to habitual baseline (*p* < 0.01), with no difference between intervention groups (*p* > 0.26). The total MVPA increased by 10 min/day during the intervention compared to the baseline (baseline: 23 ± 14 min/d vs. intervention: 33 ± 16 min/d, main effect of time *p* = 0.03, no interaction). The change in peak blood glucose (mmol/L) was similar between the ExPeak (−0.44 ± 1.6 mmol/L, *d* = 0.21) and the NonPeak (−0.39 ± 1.5 mmol/L, *d* = 0.16) intervention groups (*p* = 0.92). Prescribing an exercise time based on CGM may increase daily participation in physical activity in people with type 2 diabetes; however, further studies are needed to test the long-term impact of this approach.

## 1. Introduction

Type 2 diabetes (T2D) is characterised by hyperglycaemia with elevated fasting and postprandial glucose, largely due to insulin resistance and lower beta cell function [1]. Regular physical activity is a cornerstone therapy in the management of T2D, and the benefits for glycaemic control and cardiovascular risk are well-known. The global recommendation is to accumulate at least 150 min/week of moderate intensity aerobic activity to improve or maintain health outcomes [2], and the most common prescription is to perform 30 min of moderate-to-vigorous intensity aerobic activity most days of the week [2,3]. However, one in four adults currently do not meet the activity guidelines [2], and nearly 50% of adults with T2D do not meet the minimum amount of recommended physical activity [4]. Therefore, strategies that increase physical activity participation and/or allow individuals to make the most of their exercise (i.e., precision medicine) are urgently needed.

Growing evidence suggests that the timing of exercise is important for maximising glycaemic benefits in people with T2D. The majority of research exploring exercise timing has prescribed exercise in relation to meals (i.e., pre/post meal), a general time of day (i.e., morning/afternoon/evening) or, more recently, circadian rhythm (i.e., chronotype) [5,6,7,8,9,10]. The findings from these studies suggest exercise in the postprandial period leads to the greatest improvement in glycaemic outcomes [5,6,7], and one systematic review suggests exercising after the largest meal of the day [6]. However, T2D is a heterogenous population with different phenotypes, chronotypes, and behavioural attributes [11], which lead to variations in glycaemic responses [12] and hyperglycaemic excursions at different times of the day. Indeed, hyperglycaemic excursions in people with T2D are not limited to the postprandial period, indicating a more personalised approach is needed [13].

Continuous glucose monitoring (CGM) is emerging as a novel tool for personalised treatment in people with T2D. CGM measures interstitial glucose concentration every 5–15 min for up to 14 days, providing a dynamic assessment of individual glycaemic profiles in response to dietary intake or exercise in free-living translational conditions [12]. CGM offers additional insight into key outcomes related to cardiovascular disease, such as glycaemic variability and peak glucose concentrations [12]. As such, CGM can provide a comprehensive assessment of glucose patterns which can aid health care providers in personalising diabetes treatment plans and highlight the impact of therapies.

Exercise timing is an emerging concept, however, the likelihood of whether individuals will adhere to a prescribed exercise time and the impact of prescribing an exercise time on other physiological outcomes in free-living conditions has yet to be determined. Therefore, the aim of this study was to examine the feasibility of using CGM to prescribe personalised exercise in people with T2D. This study was not designed or powered to test the efficacy of this approach but including a non-peak time allowed for a preliminary exploration of the effect of prescribing an exercise time to target peak hyperglycaemia on short-term glycaemic outcomes in free-living people with T2D.

## 2. Material and Methods

### 2.1. Study Design

Individuals with physician-diagnosed T2D [3] were recruited via online advertising to participate in a double-blind parallel proof-of-concept study. Participants were randomised to complete one of two short-term 14 d exercise interventions, where a daily exercise time was prescribed in relation to individual time of peak hyperglycaemia determined via a habitual baseline continuous glucose monitoring (CGM) period. Informed consent was obtained from all eligible individuals prior to participation and randomisation. An automatic computer-generated random number table was used to perform random allocation of participants (1:1 ratio), stratified for sex and exogenous insulin usage, and a sealed envelope system was used to blind researchers from group allocations.

This research was reviewed and approved by the University of Wollongong Human Research Ethics Committee (HREC 2019/258).

### 2.2. Participants

Thirty-five adults with physician-diagnosed T2D, who were treated with lifestyle, oral medication, and/or intermediate/long-acting insulin with stable medication for the previous three months, were recruited. Individuals with absolute contraindications to exercise (i.e., musculoskeletal/joint injury, etc.); presence/history of cardiovascular disease (CVD), kidney, or liver disease; diagnosed diabetes complications; uncontrolled hypertension (>160/90 mmHg); treatment with short/rapid acting insulin; change in medication or weight (±4 kg) in past three months; or >150 min/week of moderate-to-vigorous intensity aerobic activity were excluded. A medical screening questionnaire (Physical Activity Readiness Questionnaire) [14] and physical activity questionnaire (Godin Leisure-Time Exercise Questionnaire) [15] were completed in order to determine eligibility.

### 2.3. Experimental Protocol

A CGM (Freestyle Libre 2, Abbott) and accelerometer (ActiGraph Bluetooth^®^ Smart wGT3X-BT) were worn during a 14 d habitual baseline period and 14 d intervention period. Data from the baseline CGM were analysed to determine the average daily individual time of peak hyperglycaemia as described in Chang et al. [16]. In brief, time of peak was determined from the ‘Glucose Pattern Insights’ report (automatically generated via LibreView software) and confirmed by calculating the average time that peak hyperglycaemia occurred on valid continuous glucose days.

The experimental group (ExPeak) were prescribed a daily exercise time, which corresponded to beginning exercise 30 min before peak hyperglycaemia, whereas the comparator group (NonPeak) were prescribed a daily exercise time to correspond with beginning exercise 90 min after the time of peak hyperglycaemia. Both groups were prescribed 22 min/d of moderate intensity aerobic activity for two weeks and were blinded to the study aim (i.e., they were not informed if they were exercising at time of peak or not at time of peak). Mode of aerobic activity (i.e., walking) was self-selected by participants and recorded during phone consultations. During the intervention period, participants wore a heart rate monitor (Polar H7 Bluetooth Heart Rate Monitor), which automatically paired with the ActiGraph accelerometer for the daily exercise sessions to measure exercise intensity. Interventions were completed in free-living conditions, with all exercise sessions unsupervised, and were undertaken at home. A phone consultation was conducted prior to the initial monitoring period and half-way through the 14 d intervention period. The first consultation addressed any questions or concerns participants had about the study protocol or testing materials. The second consultation involved discussions around exercising at the prescribed time, including the type of exercise, adherence, and barriers to the prescribed time. Questions included: (i) How have your exercise sessions been going?, (ii) How are you finding the exercise time?, (iii) Have you missed any sessions over the past week?, iiia) If yes, what barriers did you encounter preventing you from completing the exercise session?, and (iv) Do you have any other questions or concerns that you want to discuss? Other than the prescribed exercise, participants were instructed to maintain their standard lifestyle, including medication usage and dietary habits.

### 2.4. Outcomes

#### 2.4.1. Feasibility and Effects of Prescribing an Exercise Time on Physical Activity

The feasibility of this intervention was determined by exercise adherence, predefined as the proportion of participants that completed ≥ 15 min/d of moderate-to-vigorous intensity physical activity (MVPA) at the prescribed time ≥ 5 d/week. Accelerometer and heart rate data were downloaded using ActiLife version 6.13.4 Software prior to being exported to Excel for analysis. Using ActiLife software, sedentary time (minutes per week), physical activity (minutes per week of light, moderate, and moderate-to-vigorous intensity activity), and heart rate intensity (percentage of age-predicted HR-max, during exercise sessions only) were calculated. Freedson et al. [17] cut points were used to determine level of moderate intensity physical activity (≥1952 counts/min) during wear time on valid days. Participants were required to have a minimum of three valid wear days from the accelerometer, with 10 h of valid wear time each day, to be included in the analysis.

#### 2.4.2. Continuous Glucose Monitoring

Freestyle Libre data were downloaded using LibreView software prior to being exported to Excel for analysis. Mean 24 h glucose, peak glucose, and standard deviation were calculated for each full day and then averaged across the available days. The potential efficacy of the intervention was assessed by calculating the difference in average peak glucose between groups and the change from baseline.

#### 2.4.3. Dietary Intake

Dietary intake was not controlled during the habitual baseline monitoring period or the intervention; however, participants were instructed to maintain normal dietary habits and not change their diet throughout. All participants completed a seven-day food diary at baseline and during the intervention where they recorded food items, including beverages, and portion sizes at each meal and snack. Food diaries were analysed using FoodWorks 10 nutrition software to compare macronutrient composition and energy intake.

### 2.5. Statistical Analysis

This was a small proof-of-concept (remote (non-contact)/free-living) study that was conducted during the COVID-19 pandemic with no a priori sample size calculation. Descriptive statistics were assessed (means, SD, and frequencies), and histograms, Q–Q plots, and the Shapiro–Wilk test were used to identify outliers and test for normality using SPSS version 27 (IBM SPSS Statistics Inc., New York, USA). Outcomes from the CGM (24 h mean glucose, peak glucose, and standard deviation), accelerometer (total minutes of moderate-to-vigorous intensity activity), and dietary intake (carbohydrate and energy intake) were analysed with SPSS using a linear mixed model (with time x group interaction and significance set at *p* ≤ 0.05), and adherence was analysed using chi-squared tests to examine changes from baseline and between intervention groups. Data are reported as mean ± SD.

## 3. Results

Thirty-five (*n* = 35) adults with T2D were recruited and completed the intervention (baseline characteristics are shown in Table 1). Two participants were excluded for missing CGM and accelerometer data, leaving 33 for the final analyses.

### 3.1. Adherence to the Prescribed Exercise Time

**Figure 1:**
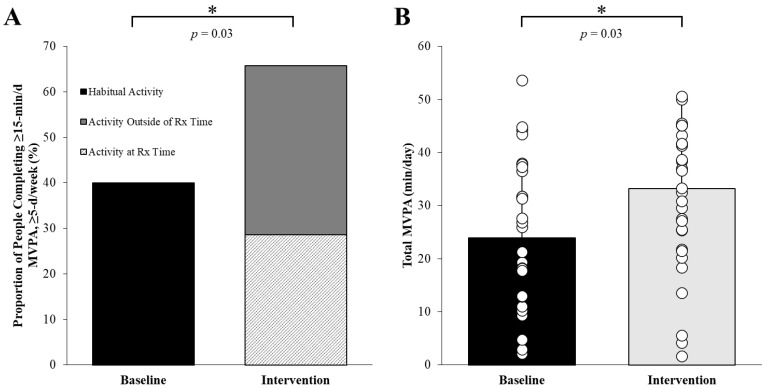
Exercise adherence (**A**) and total moderate-to-vigorous intensity physical activity (MVPA) (**B**) improved when an exercise time was prescribed. (**A**) Approximately 66% of participants completed ≥15 min of moderate intensity physical activity ≥5 days/week during the intervention compared to 40% of participants at baseline (*p* = 0.03), and (**B**) total MVPA increased by 10 min/day during the intervention compared to baseline (baseline: 23 ± 14 min/d vs. intervention: 33 ± 16 min/d, main effect of time *p* = 0.03 no interaction). * *p* = 0.03 between baseline and intervention. Values are mean ± SD, *n* = 33.

Only 29% (*n* = 8 (ExPeak) and *n* = 2 (NonPeak)) of participants consistently adhered to daily exercise at their prescribed time, while 37% (*n* = 6 (ExPeak) and *n* = 7 (NonPeak)) completed the daily exercise outside of their prescribed time, and 34% (*n* = 4 (ExPeak) and *n* = 6 (NonPeak)) did not exercise for at least 15 min ≥5 d/week (Figure 1). Overall, 66% of participants completed the prescribed exercise but not necessarily at the correct time (as discussed above).

### 3.2. Barriers to Exercise at a Prescribed Time

Participants discussed barriers to completing the prescribed exercise at the second consultation. Sixteen participants reported no issues with the prescribed time (*n* = 10 (ExPeak), *n* = 6 (NonPeak)); four did not disclose whether they were encountering any barriers (*n* = 2 (ExPeak), *n* = 2 (NonPeak)); eleven encountered scheduling conflicts due to commitments with family, friends, or work (*n* = 4 (ExPeak), *n* = 7 (NonPeak)); two were unable to exercise outdoors due to extreme weather conditions (*n* = 1 (ExPeak), *n* = 1 (NonPeak)); and two had unrelated injuries or illness (*n* = 2 (ExPeak)).

### 3.3. Impact of Prescribing an Exercise Time on Physical Activity Parameters

Participation in moderate intensity physical activity increased by 26% during the interventions compared to the habitual baseline (main effect of time: *p* = 0.03) (Figure 1), with no difference between intervention groups (interaction: *p* > 0.26). Total MVPA increased by ~10 min/d compared to the baseline (*p* = 0.03) (Figure 1), with no differences between groups (Table 2). During the intervention, exercise intensity averaged 79 ± 18% age-predicted HR-max with no difference between groups (ExPeak: 123 ± 16 bpm vs. NonPeak: 127 ± 20 bpm, *p* = 0.77).

### 3.4. Continuous Glucose Monitoring and Dietary Outcomes

**Figure 2:**
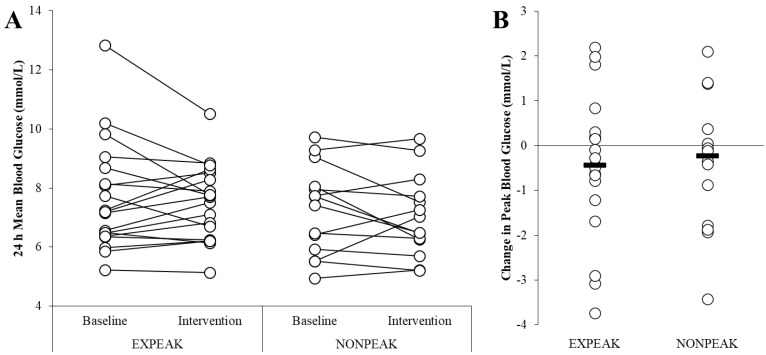
Mean and peak blood glucose levels (mmol/L) at baseline and during the two-week intervention. (**A**) Mean blood glucose was similar at baseline and during the intervention for the ExPeak (base: 7.66 ± 1.85 mmol/L, int: 7.51 ± 1.28 mmol/L, *p* = 0.56) and NonPeak (base: 7.27 ± 1.51, int: 7.03 ± 1.37 mmol/L, *p* = 0.35) groups. (**B**) The change in peak blood glucose (mmol/L) was similar between the ExPeak (−0.44 ± 1.6 mmol/L, Cohen’s d: 0.21) and NonPeak (−0.39 ± 1.5 mmol/L, Cohen’s d: 0.16) intervention groups (*p* = 0.92). Values are mean ± SD, *n* = 33.

There were no significant differences in 24 h mean glucose, peak glucose, or standard deviation (Table 2, Figure 2). Dietary outcomes were similar between groups at the baseline and during the intervention (Table 2).

## 4. Discussion

The primary aim of this proof-of-concept study was to examine the feasibility of using CGM technology to personalise the time of day to exercise in adults with T2D. The present study found that prescribing a daily exercise time, independent of whether the exercise time was aimed at reducing peak glucose or not, increased participation in total MVPA by ~10 min/day compared to the baseline physical activity levels. These findings highlight the potential of prescribing an exercise time to improve exercise habits in individuals with T2D. In addition, this strategy may, over the long term, improve health outcomes, given that a 10 min increase in MVPA per week is associated with a 15% reduction in cardiometabolic risk [18]. However, whilst prescription of an exercise time resulted in more overall MVPA, 37% of participants performed their exercise at times outside of their individually prescribed time, which may explain the lack of an effect on the change in peak glucose and other glycaemic outcomes from the CGM in the ExPeak group. Given that there is no cure for type 2 diabetes but there is a significant burden on the healthcare system, exploring and refining how existing and evolving technology can be used to help personalise medicine and improve self-monitoring is an important area of research.

Physical activity and exercise are cornerstones in T2D management. Indeed, physical activity interventions that are home-based are particularly valuable as a scalable, safe, and cost-effective frontline therapy. Despite the well-known benefits of achieving the current physical activity guidelines, we are currently facing a physical inactivity epidemic, with global activity levels remaining unchanged for the past 22 years [19]. It was unknown whether adding an exercise time would improve or worsen adherence to the physical activity guidelines. The main finding of the present study was that only 29% completed daily exercise at the prescribed time, although 66% completed the exercise prescription (achieved the recommended exercise prescription). This was significantly lower than expected, but not surprising given that only 43% [20] to 58% [21] of adults with diabetes adhere to the physical activity guidelines. Many individuals, with and without T2D, encounter barriers for completing the physical activity guidelines. In the present study, the participants briefly discussed barriers for completing the prescribed exercise at the prescribed time, and, while 46% reported no barriers with the exercise prescription, some participants stated scheduling conflicts due to other commitments (e.g., work or social) or extreme weather conditions occurring at their prescribed exercise time. Common barriers to meeting the global physical activity recommendations in individuals with T2D include co-morbidities, lack of time, extreme weather conditions, and lack of support, whereas facilitators include motivation, monitoring, encouragement, and assistance with transitioning between a supervised exercise program to a self-directed one [22]. As these barriers are similar to those reported in the current study, it is unclear whether adding a strict exercise time appears to have presented an additional barrier. Further randomised controlled trials with a control ‘exercise at any time’ condition (i.e., physical activity guidelines) are needed. Given the reported barriers in the literature, these data suggest that individuals are more likely to adhere to exercise when provided with sufficient tools and guidance to keep them accountable and motivated to exercise.

Self-regulation enables individuals to gain control over their behaviours [23,24], and one approach for improving self-regulation is to provide immediate feedback for individuals, allowing them to see the benefits of their behaviour changes [25,26]. For example, self-monitoring blood glucose (SMBG), which can be accomplished with CGM devices, is an essential self-regulatory skill for the management of blood glucose in T2D that provides immediate visual feedback on the biological effect of lifestyle choices that impact blood glucose levels [24,26]. In the current study, participants were able to see their glucose responses during the intervention period; however, they were blinded as to why they were exercising at the time that they were prescribed (i.e., they were not informed if they were exercising at time of peak or not at time of peak), and this may have contributed to the low adherence to exercising at the prescribed time. CGM can provide visual feedback, allowing individuals to observe the immediate benefits from an exercise session or the overall benefits following an exercise program completed over multiple days or weeks, which can lead to improvements in self-efficacy and adherence over the long-term [26,27]. Moreover, CGM can inform patients about how to recognise patterns in their blood glucose levels following an exercise session or a longer-term exercise program, leading to enhanced motivation and exercise adherence over time [26]. Indeed, physical activity is crucial for the successful treatment of many chronic diseases and should be included within individual treatment plans. However, many physicians abstain from prescribing exercise, which may be attributed to a perceived lack of knowledge about how to do so in a safe and effective way [19]. The findings from the current study highlight the potential of using CGM technology as a tool to improve exercise habits in people with T2D and may be a useful addition to the toolbox for practitioners. Future research should utilize an unblinded trial to test adherence and feasibility in this context.

Diabetes treatment and management is a rapidly evolving area, especially with advancing technology and monitoring. Current exercise recommendations focus on a ‘one-size-fits-all’ approach; however, it is well-known that type 2 diabetes is a heterogenous group [11], where individual characteristics can influence the effectiveness of treatment from person to person [28]. The Precision Medicine Initiative is an emerging approach in the treatment and management of T2D, which considers variability between individuals due to genetics and environmental and lifestyle influences [29]. Advances in technology largely contribute to the potential of improving precision medicine by enabling health care providers to recommend the right course of treatment for patients, at the right time, based on individual characteristics [30]. For example, these technologies, which include CGM devices, can provide detailed information about an individual’s physical condition, how they respond to certain therapies, and track the progression of disease [31], in turn providing relevant information for health care providers on how to better personalise effective treatment plans. However, the effectiveness of lifestyle treatment interventions is also largely attributed to patient adherence [23].

This is the first study to explore using CGM to prescribe an individualised daily exercise time for targeting peak glucose excursions in people with T2D. A primary strength of the current study was the use of CGM technology to individualise exercise prescription and measure glucose outcomes. In addition, accelerometer devices were used to objectively measure physical activity and adherence to exercise at a prescribed time. Due to the short duration of the intervention, we were unable to determine the efficacy of prescribing an exercise time to target glucose outcomes. Future studies are now warranted to examine the longer-term effects of using CGM to prescribe an exercise time (e.g., for glycaemic control and weight management), inclusive of individuals with varying HbA1c levels and the inclusion of strength training activities to improve the generalisability of the findings. CGMs may be a useful tool for physicians to prescribe exercise in a safe and effective way (i.e., based on the time of peak glucose concentration) due to the comprehensive assessment of the glucose patterns generated from CGM data. Given that participants were blinded to the aim of the present study (i.e., prescribing exercise at a specific time to target peak glucose excursions), future investigations are also needed to examine whether exercise adherence improves in patients when an exercise time is prescribed by their physician and when they are informed as to why their exercise is being prescribed at a specific time. Indeed, CGM technology can provide both patients and health care providers with critical information to identify and reduce hyperglycaemia in people with diabetes, thus empowering patients to strive towards achieving and maintaining optimal glycaemic control. However, the cost of CGM continues to be a limiting factor for the translation of this approach in individuals with T2D in Australia. Thus, the findings from longer-term trials may help to improve the rationale of subsidising CGM technology for individuals with T2D in Australia if the exercise prescription leads to improvements in health outcomes and/or behaviours over time.

## 5. Conclusions

This study is the first to examine whether CGM can be used to prescribe an individualised exercise time based on peak glucose concentrations in people with T2D. Prescribing an exercise time may have beneficial effects for increasing total daily MVPA, but, due to the short duration of the intervention, the longer-term glycaemic outcomes are not yet known. Given that most participants exercised outside of their prescribed time, a more achievable approach may be to give a personalised ~60 min window of time to undertake physical activity. In addition, an appropriately powered RCT including a control physical activity guideline, ‘exercise at any time’ condition, is needed. The proposed strategy is novel and translatable and a step towards precision medicine. Longer-term intervention trials are warranted to further refine and test this approach and to determine if these habits, when sustained, translate into health benefits.

## Figures and Tables

**Table 1:** Baseline characteristics of participants.

	Total	ExPeak	NonPeak
*n*	35	19	16
Age _years_	64.0 ± 7.0	65.9 ± 6.1	62.3 ± 7.4
Sex _M:F_	18:17	10:9	8:8
Insulin _ID:NID_	5:30	3:16	2:14
HbA1c _%_	7.2 ± 0.8	7.3 ± 0.8	7.1 ± 0.9
BMI _kg/m2_	29.2 ± 5.2	30.0 ± 4.7	28.2 ± 5.7

Data presented as mean ± SD. Abbreviations: ExPeak, exercise at time of peak; NonPeak, exercise not at time of peak; M, male; F, female; ID, insulin dependent; NID, non-insulin dependent; HbA1c, glycated hemoglobin; BMI, body mass index (calculated as weight in kilograms divided by height in meters squared).

**Table 2:** Physical activity, blood glucose, and dietary intake during the 14 d baseline and intervention periods.

	Baseline		Intervention		Time	Group	Interaction
	ExPeak	NonPeak	ExPeak	NonPeak	*p*-Value	*p*-Value	*p*-Value
Physical Activity							
Sedentary_min-d_	1226.2 ± 110.6	1153.1 ± 217.6	1253.8 ± 48.1	1222.7 ± 83.3	0.14	0.11	0.51
Light_min-d_	125.8 ± 50.9	117.8 ± 39.4	129.5 ± 42.0	118.1 ± 56.7	0.87	0.44	0.89
Moderate_min-d_	24.0 ± 15.0	22.7 ± 13.0	33.3 ± 16.5	31.5 ± 14.7	0.03	0.69	0.96
MVPA_min-d_	24.4 ± 15.0	23.4 ± 13.3	33.7 ± 16.9	32.5 ± 15.4	0.03	0.79	0.98
Continuous Glucose Monitoring							
Mean_mmol/L-24 h_	7.7 ± 1.9	7.3 ± 1.4	7.5 ± 1.3	7.0 ± 1.4	0.63	0.23	0.91
Peak_mmol/L-24 h_	12.4 ± 2.8	11.7 ± 2.6	11.9 ± 2.0	11.2 ± 2.3	0.43	0.22	0.97
SD_mmol/L-24 h_	1.9 ± 0.6	1.7 ± 0.6	1.7 ± 0.5	1.5 ± 0.5	0.24	0.20	0.87
Dietary Intake							
Energy_kcal-d_	2066 ± 390	1949 ± 489	1778 ± 617	1670 ± 637	0.07	0.47	0.98
PRO_%E-d_	19.6 ± 3.7	19.7 ± 4.9	17.8 ± 2.3	20.9 ± 5.2	0.80	0.17	0.20
FAT_%E-d_	32.8 ± 5.0	33.6 ± 8.2	36.5 ± 5.5	32.9 ± 8.9	0.42	0.45	0.26
CHO_%E-d_	43.0 ± 6.2	36.7 ± 15.9	41.3 ± 7.2	41.1 ± 9.4	0.62	0.23	0.25

Data presented as mean ± SD. Data analysed via linear mixed model with fixed effects for timepoint and exercise-intervention groups and the interaction between timepoint and exercise-intervention groups. Abbreviations: ExPeak, exercise at time of peak; NonPeak, exercise not at time of peak; min/d, minutes per day; MVPA, moderate-to-vigorous intensity physical activity; mmol/L, millimoles per litre over 24 h; SD, standard deviation; kcal-d, kilocalorie per day; %E-d, percentage of total energy per day; PRO, protein; CHO, carbohydrate.

## Data Availability

Data generated or analysed during this study are available from the corresponding author upon reasonable request.
